# Food insecurity: a driver of gender disparity in elevated blood pressure among adults in Ondo State, Nigeria (a cross-sectional study)

**DOI:** 10.11604/pamj.2025.52.101.46449

**Published:** 2025-11-11

**Authors:** Oluwasiji Olabisi Olaitan, Oluwaseun Ariyo

**Affiliations:** 1 Department of Food and Nutrition, Durban University of Technology, Durban, South Africa,; 2 Department of Human Nutrition and Dietetics, University of Ibadan, Ibadan, Nigeria

**Keywords:** Food insecurity, elevated blood pressure, gender, adults, Nigeria

## Abstract

**Introduction:**

food insecurity is increasing at an alarming rate, contributing to gender variation in illness and undermining the 2030 nutrition goals. Evaluating its components could aid interventions aimed at addressing gender-specific diseases such as hypertension. This study assessed gender differences in food insecurity and its association with elevated blood pressure (EBP) in Ondo State, Nigeria.

**Methods:**

this community-based cross-sectional study used a four-stage systematic random sampling to select 769 adults aged 18 years and older. Food insecurity experience scale survey module components (Worried, Healthy, Fewfoods, Skipped, Ateless, Ranout, Hungry, and Wholeday) were defined, and participants were categorized into: food secure (0 point) and food insecure (≥1 point). Obesity and abdominal adiposity were determined by body mass index (≥30kg/m^2^) and waist-height ratio (≥0.5), respectively. The EBP was defined as BP≥140/90mmHg. Multivariable logistic regression was performed at p<.05.

**Results:**

a proportion of 54.9% men and 45.1% women participated in the study. Their mean ages and monthly incomes (±standard deviation) were 32.3±11.6 years, 33.9±11.7 years, and $51.1±41.0, $46.9±37.3, respectively. Women were more food insecure (44.1%, 35.8%) and experienced Worried (34.9%, 23.9%), Healthy (34.3%, 25.8%), Fewfoods (22.8%, 18.0%), Skipped (36.0%, 26.3%), Ateless (25.4%, 20.4%), Ranout (14.4%, 10.2%), Hungry (11.8%, 10.1%) and Wholeday (5.8%, 4.5%) than men, respectively. Men (9.2%) had EBP more than women (8.6%). The EBP was associated with marital status (aOR=2.53, CI=1.09, 5.87, p=.03), worried (aOR=6.33, CI=2.25, 17.78, p=<.001), ranout (aOR=5.98, CI=1.02, 35.01, p=.04) and abdominal adiposity (aOR=2.44, CI=1.38, 5.31, p=.03) among men, but occupation (aOR=1.41, CI=1.04, 1.91, p=.03) and physical inactivity (aOR=3.63, CI=1.04, 14.30, p=0.04) among women.

**Conclusion:**

gender difference was observed in food insecurity, which significantly contributed to EBP among men, while occupation and physical inactivity predisposed women to EBP. Interventions in controlling hypertension should incorporate schemes that address lack and inadequate access to food, and maintain a healthy body weight through a quality diet and physical exercise among Nigerian adults.

## Introduction

Presently, 8.2% of the world population (673 million people) experience hunger, while 2.3 billion people are moderately or severely food insecure [1]. Africa is mostly hit by the global cases of food insecurity and malnutrition. In West and Central Africa alone, more than 49.5 million people are facing acute food insecurity [2], while Nigeria has 30.6 million people experiencing acute food insecurity and 37% population living below the poverty line [3]. Food insecurity is one of the socio-economic determinants of health, which is often neglected in the efforts targeted at curbing cardiovascular diseases in low-and middle-income countries like Nigeria [4,5]. It reflects gender inequality in socioeconomic status, access to a healthy diet, and poverty both at the individual, household, and population levels [6]. Though, the relationship between food insecurity and hypertension has been established through mental disturbance, nutritional, and physiological mechanisms. However, inconsistency in the assessment tools used limits the comparison of the findings [7-9]. Globally, women (31.9%) have been reported to be moderately and severely food insecure more than men (27.6%) [10], while men are more at risk of hypertension than women [11,12]. These disparities have been related to hormonal, physiological, and socio-economic differences [11,13]. However, there is limited information on the role of gender in the relationship between food insecurity and hypertension at the individual and population levels using a standardized measure. In lieu of this, achieving sustainable development goals (SDG) of ensuring zero hunger (SDG 2), good health and wellbeing (SDG 3) and gender equality (SDG 5) by 2030 remains blink with the prevalence of food insecurity and cardiovascular disease which continue to impede development and productivity of many low-and middle-income countries such as Nigeria where these conditions are prevalent and continue to increase treatment cost. The extent to which food insecurity contributes to or changes the course of gender differences in high blood pressure is less examined, particularly in Nigeria [14,15]. Addressing these gender-related public health problems would help in developing a population- and problem-specific intervention which can provide a long-term effect, rather than following guidelines whose application is easily influenced by socio-cultural and economic factors [16]. This study aimed to evaluate gender differences in food insecurity and identify food insecurity components that significantly contribute to gender disparity in elevated blood pressure among adults in Ondo State, Nigeria.

## Methods

**Figure 1:**
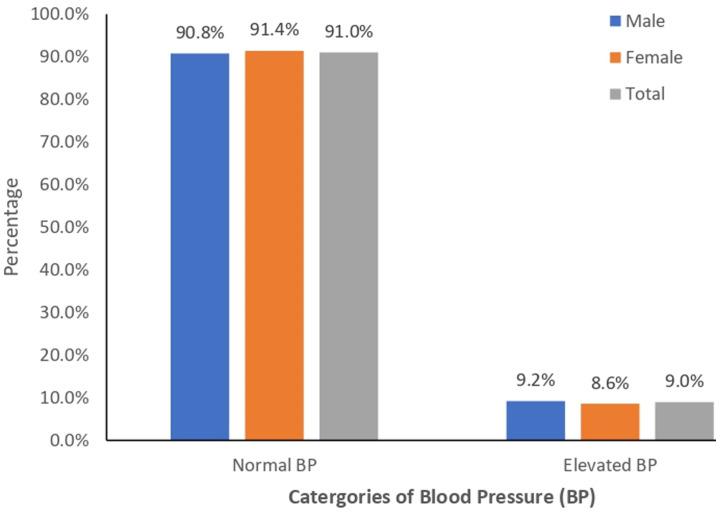
participants’ blood pressure levels

**Study design and setting:** a cross-sectional survey was conducted in Ondo State, Nigeria with the use of validated, interviewer-administered questionnaire, which was administered face-to-face to the study participants. It was a population-based study, which is useful in covering various determinants of diseases and in monitoring progress toward national, regional, and global health goals [17]. Ondo State is one of the six states in the western region of Nigeria. It was established on February 3^rd^ 1976. Its capital city is Akure. It has three (3) senatorial districts (Ondo North, South, and Central) and 18 government areas (LGAs). The indigenes of Ondo State are grouped according to their local dialects, which include: Akoko, Owo, Ondo, Ikale, Ilaje, Akure, Ijaw-Arogbo and Ijaw Apois. The estimated population of Ondo State is about 3,460,877. The state borders Ekiti, Kogi, Delta, Ogun, and Osun states to the north, north-east, south-east, south-west, and north-west, respectively (Figure 1) [18].

**Study population:** male and female adults who were within the age of 20-60 years and gave informed consents to participate in the study were recruited by a four-stage random systematic sampling. The choice of the age group was based on the report of previous studies which have confirmed that high blood pressure is more prevalent among adults aged 20 years and older in Nigeria. The age group also represents an active and productive population that contributes to the country´s economy [19]. Out of 18 local government areas (LGAs) in Ondo State, six (6) LGAs (Owo and Akoko South East, Okitipupa and Ilaje, Akure South, and Ondo West) were selected using systematic random sampling, having two LGAs representing each senatorial district (Ondo North, South and Central). From the ward of each LGA, selection of households with eligible male and female adults in each ward was identified and listed. Household interval was calculated based on the number of respondents who were eligible.

**Data collection:** an interviewer-administered questionnaire was developed to assess sociodemographic characteristics and lifestyle practices. Food insecurity was determined by using the eight-point food insecurity experience scale survey module (FIES-SM), and categorized into food secure (0 point), and food insecure (≥1 point) [20,21]. Body weight and height were measured by a bathroom weighing scale and a stadiometer, respectively. Body mass index (kg/m^2^) was calculated, and obesity was defined as body mass index (BMI) of ≥30.0kg/m^2^, respectively. Abdominal adiposity was determined by the waist-to-height ratio, and was established at 0.5 point and above for both genders [22]. A clinically validated monitor with the label of Omron M2 Eco (HEM-7120-AF) was used to assess blood pressure following a protocol developed by the International Society of Hypertension [23]. The measuring instruments were pretested through a pilot study to determine the validity and reliability before commencement of the survey.

**Definitions:** a short form of the International Physical Activity Questionnaire was adapted to assess the level of respondents´ physical activity. Performance of physical activity in a week below 600 metabolic equivalent minutes per week (MET-min/week) was considered as physical inactivity [24]. Eight-point food insecurity experience scale survey module (FIES-SM) was developed by the United Nations Food and Agriculture Organization to measure a person´s behaviours related to food consumption and experience associated with difficulty in food access due to constraints of resources in the preceding 30 days. It is based on “Yes” or “No” responses to eight sets of questions, which are abbreviated as: Worried, Healthy, Fewfoods, Skipped, Ateless, Ranout, Hungry, and Wholeday, the food insecurity components [20,21]. Waist circumference and height were measured by a non-stretchable measuring tape and stadiometer, respectively. Waist circumference (cm) was divided by height (cm) to calculate the waist-to-height ratio. Blood pressure (BP) of each respondent was measured by placing the cuff on the left mid-arm. Three blood pressure readings were taken with an interval of 1 to 2 minutes between each measurement. The last two readings were summed up, and their mean was used as the actual value. Elevated blood pressure was defined as systolic and diastolic blood pressures equal to or greater than 140mmHg and 90mmHg, respectively [23].

**Statistical analysis:** data were analysed by using Statistical Product and Service Solution (SPSS) version 29.0. Means, standard deviation, frequencies, and percentages were descriptive statistics carried out. Univariable analysis was performed through cross-tabulation and chi-square, while multivariate binary logistic regression was conducted to determine factors that significantly contribute to the odds of developing elevated blood pressure and to account for confounders. The outcome variable is elevated blood pressure, while food insecurity is the independent variable. Level of statistical significance was set at p<.05.

**Ethical considerations:** ethical approval for this study was granted by the research ethics committee of the University of Ibadan College Hospital, Ibadan, bearing reference number UI/EC/21/0110. Voluntary participation and informed consent were obtained from the study participants before commencing data collection. Participants´ information was deidentified with codes to ensure the confidentiality of the participants.

## Results

**General characteristics of the study population:** Table 1 presents information on the general characteristics of the study participants. A total of 769 adults (54.9% males, 45.1% females) participated in the study, with the mean ages of 32.3±11.6 years and 33.9±11.7 years, respectively. More than half of the respondents (55.7%) were within the age of 20 to 30 years (58.5% men, 55.7% women), 51.7% were married (44.8% men, 59.9% women), 55.3% had attained formal education up to secondary level (56.2% men, 54.2%) and 52.9% worked either as artisans (21.1% men, 22.2% women) or traders (26.8%, 36.9%) and earned between $28.47 and $56.94 in a month. Out of 23.0% who drank alcohol, 30.8% were men, while 13.5% were women. Those who smoked cigarettes were 4.7% (8.1% men, 0.6% women). “Overall, 27.8% of respondents were physically inactive, with a higher prevalence among women (30.5%) than men (25.6%).

**Table 1:** general Information of study population

Variables	Total	Male	Female
	769 (100%)	422 (100%)	347 (100%)
**Age (years)**			
20-30	428 (55.7)	247(58.5)	181 (52.2)
31-40	146 (19.0)	71 (16.8)	75 (21.6)
41-50	112(14.6)	61 (14.5)	51(14.7)
51-60	83(10.8)	43(10.2)	40 (11.5)
Mean ± SD	33.1±11.6	32.3±11.6	33.9±11.7
**Marital status**			
Single	349(45.4)	227(53.8)	122(35.2)
Married	397(51.7)	189(44.8)	208(59.9)
Divorced/separated	10(1.3)	6(1.4)	4(1.2)
Widowed	13(1.7)	0(0.0)	13(3.7)
**Education attainment**			
Primary	86(11.2)	35(8.3)	51(14.7)
Secondary	425(55.3)	237(56.2)	188(54.2)
Tertiary	248(32.2)	145(34.4)	103 (29.7)
No formal education	10(1.3)	5(1.2)	5(1.4)
**Occupation**			
Artisans	166(21.6)	89 (21.1)	77(22.2)
Traders	241 (31.3)	113 (26.8)	128(36.9)
Civil Servants	71(9.2)	45(10.7)	26(7.5)
Students/Unemployed	229(29.8)	135(32.0)	94 (27.1)
Farmers	42(5.5)	22(5.2)	20(5.8)
Others (Clergy, retirees)	20(2.6)	18(4.3)	2(0.6)
**Monthly income ($)**			
<28.47	232 (30.2)	123(29.1)	109(31.4)
28.47-56.94	388(50.5)	205 (48.6)	183(52.7)
>56.94	149(19.4)	94(22.3)	55(15.9)
Mean (SD)	49.2±39.42	51.1±41.0	46.9±37.3
**Alcohol intake**			
Drinking	177(23.0)	130(30.8)	4(13.5)
Non-drinking	592(77.0)	292(69.2)	300 (86.5)
**Cigarette smoking**			
Smokers	36 (4.7)	34 (8.1)	2 (0.6)
Non-smokers	733 (95.3)	388 (91.9)	345 (99.4)
**Physical activity**			
Active	555 (72.2)	314 (74.4)	241(69.5)
Inactive	214 (27.8)	108 (25.6)	106 (30.5)

**Nutritional status, food insecurity and elevated blood pressure of the participants:** prevalence of overweight (28.2%), obesity (8.7%), and abdominal adiposity (41.7%) was significantly higher among women (31.7%, 14.1%, 54.8%) than men (25.4%, 4.3%, 31.0%), respectively (Table 2). The rate of food insecurity was 39.5%, which was significantly higher among women (44.1%) than men (35.8%), (X^2^=5.50, p=.02). Of all the food insecurity components assessed, the proportions of women (34.9%, 34.3% and 26.3%) who worried (X^2^=11.09, p=<.01), who did not eat healthy foods (X^2^=6.54, p=.01) and who skipped foods (X^2^=8.46, p=<.01) were significantly higher than men (23.9%, 25.8% and 26.3%), respectively (Table 3). Prevalence of elevated blood pressure was 9.0%, and it was higher among men (9.2%) than women (8.6%), X^2^= 0.08, p=.77 (Figure 1).

**Table 2:** anthropometric indices of participants

Parameters	Total	Male	Female	X2	P-value
	769 (100%)	422(100%)	347 (100%)		
**BMI (kg/m2)**					
Underweight	34(4.4)	20 (4.7)	14 (4.0)	32.33	<0.01*
Normal weight	451(58.6)	277 (65.6)	174 (50.1)		
Overweight	217 (28.2)	107 (25.4)	110 (31.7)		
Obese	67 (8.7)	18 (4.3)	49 (14.1)		
**Waist-height ratio**					
Normal	448 (58.3)	291(69.0)	157(45.2)	19.36	<0.01*
Elevated	321(41.7)	131(31.0)	190(54.8)		

* Statistically significant at p<.05 ;BMI:body mass index

**Table 3:** participants’ responses to FIES-SM questions

Variables	Total	Male	Female	X2	P-value
	769 (100%)	422 (100%)	347 (100%)		
**Worried**					
Yes	222(28.9)	101(23.9)	121(34.9)	11.09	
No	547(71.1)	32(76.1)	22(65.1)		<0.01*
**Healthy**					
Yes	228(29.6)	10(25.8)	119(34.3)	6.54	
No	541(70.4)	313(74.2)	228(65.7)		0.01*
**Few Foods**					
Yes	155(20.2)	76(18.0)	79(22.8)	2.68	
No	614(79.8)	346(82.0)	268(77.2)		0.10
**Skipped**					
Yes	236(30.7)	111(26.3)	125(36.0)	8.46	
No	533(69.3)	311(73.7)	222(64.0)		<0.01*
**Ateless**					
Yes	174(22.6)	86(20.4)	88(25.4)	2.69	
No	595(77.4)	336(79.6)	259(74.6)		0.10
**Ran out**					
Yes	93(12.1)	43(10.2)	50(14.4)	3.19	
No	676(87.9)	379(89.8)	297(85.6)		0.07*
**Hungry**					
Yes	78(10.1)	37(8.8)	41(11.8)	1.94	
No	691(89.9)	385(91.2)	306(88.2)		0.16
**Whole day**					
Yes	39(5.1)	19(4.5)	20(5.8)	0.63	
No	730(94.9)	403(95.5)	327(94.2)		0.51
**Level of food insecurity**					
Food secure	465(60.5)	271(64.2)	194(55.9)	5.50	
Food insecure	304(39.5)	151(35.8)	153(44.1)		0.02*

* Statistically significant at p<.05

**Factors associated with elevated blood pressure among participants:** in Table 4, factors that significantly associated with elevated blood pressure among study participants are presented. These factors include marital status (X^2^= 30.06, p=<0.01), occupation (X^2^= 23.35, p=<.01), “worried” (X^2^= 28.23, p=<.01), “healthy” (X^2^= 8.48, p=<.01), few foods (X^2^= 4.98, p=.03), skipped (X^2^= 16.45, p=<.01), “ate less” (X^2^= 3.71, p=.05), ran out (X^2^= 6.63, p=.01), hungry (X^2^= 6.29, p=0.01), whole day (X^2^= 6.69, p=.01), obesity (X^2^= 9.78, p=<.01), abdominal adiposity (X^2^= 19.36, p=<0.01), physical inactivity (X^2^= 6.71, p=.01) and food insecurity (X^2^= 23.35, p=<.01).

**Table 4:** factors associated with elevated blood pressure among participants

Variables	Elevated	Normal	X2	P-value
	BP 69 (9.0%)	BP 700 (91.0%)		
**Gender**				
Male	39(5.1)	383(49.8)	0.08	
Female	30 (3.9)	317 (41.2)		0.77
**Marital status**				
Single	11(1.4)	338(44.0)	30.06	
Married	56(7.3)	341(44.3)		
Divorced/separated	2(0.3)	8(1.0)		
Widowed	0(0.0)	13(1.7)		<0.01*
**Occupation**				
Artisans	14(1.8)	152(19.8)	23.35	
Traders	37(4.8)	204(26.5)		
Civil servants	3(0.4)	68(8.8)		
Students/unemployed	8(1.0)	221(28.7)		
Farmers	4(0.5)	38(4.9)		
Others (Clergy, retirees)	3(0.4)	17(2.2)		<0.01*
**Worried**				
Yes	39(5.1)	183(23.8)	28.23	
No	30(3.9)	517(67.2)		<0.01*
**Healthy**				
Yes	31(4.1)	197(25.6)	8.48	
No	38(4.9)	503(65.4)		<0.01*
**Few foods**				
Yes	21(2.7)	134(17.4)	4.98	
No	48(6.2)	566(73.6)		0.03*
**Skipped**				
Yes	36(4.7)	200(26.0)	16.45	
No	33(4.3)	500(65.0)		<0.01*
**Ateless**				
Yes No	22(2.9) 47(6.1)	152(19.8) 548(71.3)	3.71	0.05*
**Ran out**				
Yes	15(2.0)	78(10.1)	6.63	0.01*
No	54(7.0)	622(80.9)		
**Hungry**				
Yes	13(1.7)	65(8.5)	6.29	
No	56(7.3)	635(82.6)		0.01*
**Whole day**				
Yes	8(1.0)	31(4.0)	6.69	0.01*
No	61(7.9)	669(87.0)		
**Obesity**				
Obese	13(1.7)	54(7.0)	9.78	
Non-obese	56(7.3)	646(84.0)		<0.01*
**Abdominal adiposity**				
Obese	46(6.0)	275(35.8)	19.36	
Normal	23(3.0)	425(55.3)		<0.01*
**Physical inactivity**				
Active	59(7.7)	204(26.5)	6.71	
Inactive	10(1.3)	496(64.5)		0.01*
**Food insecurity**				
Food secure	23(3.0)	442(57.5)	23.35	
Food insecure	46(6.0)	258(33.6)		<0.01*

* Statistically significant at p<.05; Bp:blood pressure

**Gender differences in elevated blood pressure among participants:** generally, worried about the food to eat (aOR: 2.95, 95% CI: 1.36-6.42; p= 0.01), abdominal adiposity (aOR: 2.18, 95% CI: 1.22-3.88; p= 0.01) and physical inactivity (aOR: 2.25, 95% CI: 1.09-4.62; p= 0.03) were found significant factors that contributed to the elevated blood pressure among the study participants. However, in comparing men to women, marital status (aOR: 2.53, 95% CI: 1.09-5.87; p= 0.03), “worried about food” (aOR: 6.33, 95% CI: 2.25-17.78; p= <0.01), “ran out of food” (aOR: 5.98, 95% CI: 1.02-35.01; p= 0.04) and abdominal adiposity (aOR: 2.44, 95% CI: 1.12-5.31; p= 0.03) were factors significantly predisposing men to elevated blood pressure while occupation (aOR: 1.41, 95% CI: 1.04-1.91; p= 0.03) and physical inactivity (aOR: 3.86, 95% CI: 1.04-14.30; p= 0.04) significantly predisposed women to elevated blood pressure (Table 5).

**Table 5:** factors contributing to gender differences in elevated blood pressure among participants

Variables	Total			Male			Female		
	aOR	95% CI	p	aOR	95% CI	p	aOR	95% CI	p
Marital status	1.50	0.93, 2.43	0.09	2.53	1.09, 5.87	0.03*	1.46	0.71, 3.00	0.30
Occupation	1.08	0.88, 1.32	0.46	0.83	0.61, 1.13	0.24	1.41	1.04, 1.91	0.03*
Worried	2.95	1.36, 6.42	0.01*	6.33	2.25, 17.78	<0.01*	1.08	0.30, 3.83	0.91
Healthy	0.42	0.14, 1.25	0.12	0.25	0.05, 1.22	0.09	1.03	0.18, 5.99	0.98
Few Foods	0.80	0.22, 2.94	0.74	0.47	0.06, 3.63	0.47	1.71	0.30, 9.67	0.54
Skipped	2.75	0.95, 8.01	0.06	3.59	0.79, 16.15	0.09	2.19	0.36, 13.26	0.39
Ateless	0.67	0.19, 2.30	0.52	0.29	0.04, 2.10	0.23	0.66	0.12, 3.72	0.64
Ranout	1.08	0.34, 3.44	0.89	5.98	1.02, 35.01	0.04*	0.37	0.07, 1.89	0.23
Hungry	0.94	0.27, 3.32	0.92	0.67	0.10, 4.53	0.69	1.21	0.18, 8.12	0.84
Whole day	2.42	0.67, 8.73	0.18	0.71	0.09, 5.52	0.74	5.65	0.83, 38.59	0.08
Obesity	1.49	0.70, 3.17	0.29	1.41	0.35, 5.69	0.63	2.17	0.82, 5.72	0.12
Abdominal adiposity	2.18	1.22, 3.88	0.01*	2.44	1.12, 5.31	0.03*	1.79	0.66, 4.86	0.25
Physical inactivity	2.25	1.09, 4.62	0.03*	1.88	0.74, 4.78	1.89	3.86	1.04, 14.30	0.04*

*Statistically significant at p<0.05

## Discussion

Gender differences in food insecurity and food insecurity components, which significantly contribute to gender disparity in elevated blood pressure among adults in Ondo State, Nigeria are established by this study. The findings of this study show gender disparity in the level of food insecurity among the study participants. Women were found to be more food insecure than men. The observation of this study corroborates the reports from previous studies, which reported gender differences in food insecurity and its relationship with inequality in household income and social networking, level of formal education, and cultural taboos that limit women´s access to sufficient, safe, and nutritious food more than men [6,25]. Finding higher prevalence of food insecurity among women in this study can be attributed to their low socioeconomic status. When compared to men, women had a low level of formal education, were traders and artisans, and earned a low monthly income. These socioeconomic factors are essential in securing consistent and quality food [25]. Women´s limited access to resources in Africa reduces their purchasing power and access to healthy and nutritious foods [6]. However, observation on gender differences in elevated blood pressure shows that men had elevated blood pressure more than women. Previous studies have demonstrated that sex hormones and physiological differences play key roles in gender differences in cardiovascular diseases [11,13]. Oestrogen during premenopausal age and positive attitude of women towards healthcare have been reported to lower their risk of hypertension [19,26]. In relation to food insecurity, “worried” and “ran out” were food insecurity components that significantly predisposed men to elevated blood pressure. The relationship between food insecurity and cardio-metabolic conditions has been illustrated by the link of psychological disturbance and malnutrition with cardiovascular wellbeing [5,27].

Lack or insufficient amount of food at the right time and running out of food without certainty of getting food predisposes to mental stress [28,29]. Studies have shown that food insecurity is related to depression, sleep disorders, and anxiety, which are mental illnesses, and the risk factors of high blood pressure through autonomic dysregulation and hormonal disruption [7-9]. Food-insecure individuals seek cheap foods, which are mostly poor in quality, containing high salt, saturated fat, and refined sugar, which are risk factors of elevated blood pressure and other metabolic diseases [4,23]. Additionally, people who are food insecure tend to neglect seeking healthcare services. They forgo medication and result to self-management of ailment to deal with pressing needs, which oftentimes is food [30]. However, women were more predisposed to elevated blood pressure as a result of their occupation and physical inactivity. Majority of the women in the study location were either traders or artisans whose nature of work requires sitting for long periods of time. Studies have reported that sedentary behaviour and physical inactivity have been reported as major occupational risk factors of hypertension [31]. While this study establishes the gender differences in the experience of food insecurity and its contribution to elevated blood pressure in each gender, the cross-sectional nature of the design of the study limits its evaluation of the causal relationship between risk factors and elevated blood pressure among the study participants.

## Conclusion

The findings of this study show a gender difference in food insecurity and elevated blood pressure. Though food insecurity is more predominant among women, it did not significantly contribute to the risk of developing elevated blood pressure among them. Occupation and physical inactivity predisposed women to elevated blood pressure. However, food insecurity as well as abdominal adiposity contribute to the elevated blood pressure experienced by men.

### What is known about this topic

- Gender differences in food insecurity and elevated blood pressure have been established; contribution of food insecurity to hypertension has been elucidated.

### What this study adds

- Gender difference in the factors predisposing to elevated blood pressure in a given population is established;
- Mechanism through which food insecurity contributes to the elevated blood pressure is established using standardized measure, the food insecurity experience scale module.
